# A new insight into male fertility preservation for patients with completely immotile spermatozoa

**DOI:** 10.1186/s12958-017-0294-x

**Published:** 2017-09-18

**Authors:** Huanhua Chen, Guixue Feng, Bo Zhang, Hong Zhou, Caizhu Wang, Jinhui Shu, Xianyou Gan, Ruoyun Lin, Dongmei Huang, Yingqin Huang

**Affiliations:** Reproductive Medicine Center, Maternal and Child Health Hospital of Guangxi Zhuang Autonomous Region, Nanning, 530003 China

**Keywords:** Sperm cryopreservation, Completely immotile sperm, Intracytoplasmic sperm injection, Laser-plus

## Abstract

**Background:**

Sperm cryopreservation is the most effective method to preserve male fertility but this is normally used for motile spermatozoa. Thus, only motile spermatozoa are used for cryopreservation in most reproductive medicine centers worldwide. The immotile spermatozoa from some problematic patients are usually discarded, resulting in a missed opportunity of sterility cryopreservation for future assisted reproductive treatments. Many studies have shown that successful fertilization can be obtained after selection of viable sperm from the completely immotile spermatozoa before ICSI. Whether the completely immotile spermatozoa are worth of freezing has not been realized The aim of this study is to explore the clinical value of cryopreservation of immotile spermatozoa.

**Methods:**

Completely immotile spermatozoa were collected and frozen, and subsequently viable but immotile frozen-thawed spermatozoa were selected by laser plus for ICSI. Main outcomes included spermatozoa survival index, fertilization rate and good quality embryo rate.

**Results:**

After identification by laser, the fresh samples of spermatozoa presented with a mean survival rate of 54.86% and 26.05%, and this was reduced to 44.13% and 18.13% in frozen-thawed spermatozoa samples, which showed a frozen-thawed spermatozoa survival index of 0.80 and 0.70 in the testicular and ejaculate sperm, respectively. There were no statistically differences in fertilization rate (80% vs80.51%, 75.00% vs 81.48%), cleavage rate (95.45% vs 98.95%, 100.00% vs 95.45%) and good quality embryo rate (40.48% vs 52.13%, 33.33%vs38.10%) between the frozen-thawed immotile spermatozoa group and the routine fresh immotile spermatozoa ICSI group in both testicular and ejaculate sperm, respectively.

**Conclusions:**

The results of the study show that completely immotile spermatozoa can be frozen in order to preserve male fertility as long as viable spermatozoa are present. This procedure provides a further possibility for fertility preservation for patients with completely immotile spermatozoa.

## Background

Intracytoplasmic sperm injection (ICSI) has been an ideal tool to treat severe male infertility. It was reported that ICSI using completely immotile sperm resulted in successful clinical outcomes and healthy offspring [1–4]. However, this resulted in lower fertilization rate, lower embryo development potential [5] and even a higher failure-fertilization rate compared to the use of motile sperm [6, 7]. As is well known, completely immotile sperm does not mean dead sperm. Successful fertilization were observed after selection of viable sperm from completely immotile sperm before ICSI [8, 9], which suggests that the basis of ICSI requires a live spermatozoon and not one that has to be motile.

Sperm cryopreservation is the most effective way to preserve male fertility, which becomes one of the most important aspects of assisted reproductive technology (ART). Many studies showed that similar clinical outcomes were achieved after ICSI using the frozen-thawed motile sperm from an ejaculate [10, 11], percutaneous epididymal sperm aspriration (PESA) [12, 13] and testicular aspiration (TESA) [14–16] as compared to that of fresh motile sperm. In clinical practice, only motile spermatozoa, including those from ejaculate, PESA and TESA, are used for cryopreservation in most centers worldwide [17], which suggests that a prerequisite of sperm cryopreservation are motile sperms. However, the immotile spermatozoa from some problematic patients are usually discarded, resulting in a missed opportunity of sterility cryopreservation for future assisted reproductive treatments. The potential usefulness of completely immotile sperm for cryopreservation has not been realized.

The key to explore the value of cryopreservation of complete immotile spermatozoa is how to identify and select viable spermatozoa before and after cryopreservation. It is well established that laser plus is an effective method to identify viable but immotile sperm, and showed great merits over conventional methods, including hypo-osmotic swelling (HOS) and eosin staining [9, 18, 19]. The present study was undertaken to explore the clinical value of cryopreservation of complete immotile spermatozoa by laser assisted selection.

## Methods

### Patients

Thirty-two patients with completely immotile sperm were enrolled in this study, including seven with ejaculated samples and twenty-five with testicular sperm samples from March 2015 to August 2017. The sex hormone levels and the *chromosome karyotype* of each patient were considered to be normal. The results of semen analysis according to the fifth edition of World Health Organization (WHO) laboratory manual for the examination and processing of human semen guidelines [20] repeatedly showed a severe asthenozoospermia with a complete absence of motile spermatozoa in the seven patients with ejaculated sperm samples. The diagnosis of asthenozoospermia was defined as the ratio of forward motile sperm less than 32% according to the WHO laboratory manual for the examination and processing of human semen guidelines [20]. TESA were performed for the other twenty-five patients with azoospermia in order to retrieve testicular sperm for ICSI. Azoospermia is defined as no sperm was found in the precipitate of ejaculated semen sample after centrifugation according to the fifth edition of WHO laboratory manual. We considered the testicular sperm were immotile after observation of almost the whole sperm sample under an inverted microscope (×200 magnification) while none of a motile sperm was found.

### Sperm viability assessment before cryopreservation

Sperm viability was assessed by a direct laser to the tip of the sperm flagellum according to the method of Aktan et al. [21]. Briefly, the tip of completely immotile sperm were shot using the RI Saturn Laser System (England) by a laser beam of approximately 200 μJ with about 2 ms of irradiation time. The spermatozoon, presenting a curling of the tail after laser shot, was regarded as viable. In our study, the viability rate of one sperm sample was detected before (pre-freezing survival rate) and after (post-freezing survival rate) freezing, respectively. Cryopreservation was performed immediately after testing the pre-freezing survival rate.

### Sperm frozen-thawed protocols

The frozen-thawed protocol used was that established by Desch [22]. Briefly, Sperm sample in a 200uL suspension was concentrated into 5 μL by centrifugation for 3 min at 1000 rpm, then diluted with a sperm cryoprotectant medium (SAGE, USA) in a 1:1 ratio. After being maintained at the liquid nitrogen surface for 4 min, the cryogenic vial (Corning Incorporated, Mexico) was rapidly immersed in liquid nitrogen at −196 °C for long-term cryopreservation.

For the thawing procedure, firstly, the cryogenic vial was taken out of the liquid nitrogen, and placed into a 37 °C water bath immediately and kept for 15 min before transferring to a Quinn’s 1020 (SAGE, USA) operation dish.

### Sperm viability assessment after cryopreservation

The primary completely immotile sperm after cryopreservation were washed by centrifugation for 5 min at 1500 rpm, and then a pellet containing the sperm was added to a Quinn’s-1020 operation dish. Sperm viability was also assessed by a direct laser shot to the tip of the sperm flagellum as described above. Then the index of sperm survival rate which defined as the post-frozen survival rate divided by the pre-freezing survival rate was calculated to explore the freezing effectiveness of immotile sperm.

### Validation of fertilization ability after frozen-thawed treatment

Subsequently, the viable sperm were selected for injection into donated mature oocytes in order to confirm the fertilization ability of frozen-thawed immotile sperm. A total of 75 donated mature oocytes were from those patients who had achieved normal fertilization (fertilization rate was more than 80%) in their routine in vitro fertilization (IVF) cycles. So, we speculated that all of mature oocytes of these patients should have normal fertilization competence. Sometimes, we found that a few of mature oocytes were not fertilized on the day of pronucleus checking which may due to incompletely mature on the day of fertilization. Therefore, these surplus mature oocytes were collected to verify the fertilization competence of frozen-thawed immotile spermatozoa after fully informed consent. Viable but immotile frozen-thawed sperm were selected by laser for injection into these donated mature oocytes. Normal fertilization ability using the frozen-thawed immotile sperm was confirmed if two pronuclei and two polar bodies were observed at 16-18 h after ICSI. These results were compared with those of routine ICSI which were obtained from the couples who performed assisted reproductive treatments using their fresh immotile sperm.

### Fertilization checking, embryo culture and assessment

Fertilization was checked by meaning detection of two pronuclei and two polar bodies at 16–18 h after ICSI. Quinn’s medium was used for embryo culture. Zygotes displaying two pronuclei were transferred to the Quinn’s 1026 medium (Sage, USA) supplemented with 10% serum protein substitute (SPS, Sage, USA) to culture to day 3. The day of ICSI manipulation was considered as day 0. Embryo assessment is based on morphology according to the Istanbul consensus workshop on embryo assessment [23]. Briefly, day 3 cleavage embryos with stage-specific cell size, great than or equal to 6 cells, and <20% fragmentation were regarded as good quality embryos.

### Statistical analysis

Statistical analysis was completed with Statistical Package for Social Sciences version 13.0 (SPSS, USA). Proportional data in groups were compared in chi-squared analysis. Statistical significance was attributed to two-tailed *P* < 0.05.

## Results

### Effectiveness of cryopreservation of completely immotile sperm

After identification by laser, the fresh samples of spermatozoa presented with a mean survival rate of 54.86% and 26.05%, and this was reduced to 44.13% and 18.13% in frozen-thawed spermatozoa samples, which represented a frozen-thawed spermatozoa survival index of 0.80 and 0.70 in the testicular and ejaculate sperm, respectively (Tables 1 and 2).

**Table 1:** Comparison of the survival rate of completely immotile testicular spermatozoa before and after cryopreservation

Patients	Before cryopreservation			After cryopreservation			Survival index
	No. of sperm (n)	No. of live sperm (n)	Survival rate (%)	No. of sperm (n)	No. of live sperm (n)	Survival rate (%)	
1	104	71	68.27	111	75	65.57	0.99
2	109	94	86.24	120	79	65.83	0.76
3	118	67	56.78	115	58	50.43	0.89
4	130	93	71.54	115	73	63.48	0.89
5	121	69	57.02	46	18	39.13	0.69
6	107	61	57.01	117	55	47.01	0.82
7	87	48	55.17	74	26	35.14	0.64
8	104	58	55.76	108	43	39.81	0.71
9	105	63	60.00	81	38	46.91	0.78
10	109	60	55.05	102	36	35.29	0.64
11	104	58	55.75	108	43	39.81	0.71
12	119	64	53.78	136	48	35.29	0.66
13	136	76	55.88	117	50	42.74	0.76
14	100	50	50.00	83	36	43.37	0.87
15	143	87	60.84	132	73	55.30	0.91
16	135	62	45.93	119	44	36.97	0.80
17	113	57	50.44	121	47	38.84	0.77
18	122	51	41.80	108	34	31.48	0.75
19	134	62	46.27	116	42	36.21	0.78
20	129	75	58.14	131	65	49.62	0.85
21	145	81	55.86	140	68	48.57	0.87
22	154	83	53.90	146	65	44.52	0.83
23	137	59	43.07	122	39	31.97	0.74
24	131	57	43.51	120	46	38.33	0.88
25	121	49	40.50	106	32	30.18	0.75
Total	3017	1655	54.86	2794	1233	44.13	0.80

**Table 2:** Comparison of the survival rate of completely immotile ejaculate spermatozoa before and after cryopreservation

Patients	Before cryopreservation			After cryopreservation			Survival index
	No.of sperm (n)	No. of live sperm (n)	Survival rate (%)	No. ofsperm (n)	No. of live sperm (n)	Survival rate (%)	
1	230	54	23.48	204	21	10.29	0.45
2	126	30	23.80	115	15	13.04	0.55
3	246	54	21.95	238	38	15.97	0.73
4	213	62	29.11	225	52	23.11	0.79
5	245	84	34.29	229	63	27.51	0.80
6	228	45	19.74	241	29	12.03	0.61
7	236	68	28.81	215	48	22.33	0.78
Total	1524	397	26.05	1467	266	18.13	0.70

### Validation of fertilization capacity of primary completely immotile sperm after cryopreservation

As showed in Table 3, a total of 75 donated mature oocytes were injected using primary completely immotile testicular and ejaculate sperm after cryopreservation. There were no statistically differences in fertilization rate (80% vs80.51%, 75.00% vs 81.48%), cleavage rate (95.45% vs 98.95%, 100.00% vs 95.45%) and good quality embryo rate (40.48% vs 52.13%, 33.33% vs 38.10%) between the frozen-thawed immotile spermatozoa group and the routine fresh immotile spermatozoa ICSI group in both testicular and ejaculate sperm, respectively.

**Table 3:** Comparison of the normal fertilization rate and embryo developmental competence after ICSI using fresh and frozen-thawed completely immotile spermatozoa

	testicular samples		*P*	ejaculated samples		*P*
	Fresh immotile sperm	Frozen-thawed immotile sperm		Fresh immotile sperm	Frozen-thawed immotile sperm	
No .of MII oocytes	118	55		27	20	
Fertilization rate	80.51% (95/118)	80.00% (44/55)	>0.05	81.48% (22/27)	75.00% (15/20)	>0.05
Oocyte degeneration rate	0.00% (0/95)	0.00% (0/55)	–	0.00% (0/27)	0.00% (0/20)	–
Cleavage rate	98.95% (94/95)	95.45% (42/44)	>0.05	95.45% (21/22)	100.00% (15/15)	>0.05
Good quality embryo rate	52.13% (49/94)	40.48% (17/42)	>0.05	38.10% (8/21)	33.33% (5/15)	>0.05

Frozen-thawed immotile sperm groupd were used donated oocytes

## Discussion

The present study was undertaken to investigate the clinical value of cryopreservation of primary completely immotile sperm, including ejaculate and testicular sperm, and we found that high survival rates with a total survival index of 0.80 and 0.70 were obtained in the testicular and ejaculate sperm, respectively. Furthermore, we also confirmed that ICSI using the frozen-thawed viable sperm from primary completely immotile sperm also resulted in a normal fertilization and similar embryo development competence as compared to fresh immotile sperm groups. Our results may provide a new approach for the preservation of fertility in patients with completely immotile spermatozoa.

Sperm cryopreservation is the most effective way to preserve male fertility, and therefore it is an important part in ART. Regarding the cryodamage to sperm, only motile sperm is supposed to be cryopreserved in routine clinical procedures [22], while the immotile spermatozoa is often discarded, resulting in a lost chance of assisted reproductive treatment for some patients. However, studies have shown that immotile spermatozoa did not necessarily mean dead sperm. It appears that some of the immotile spermatozoa are still viable, and when presented with a normal fertilization possibility is able to produce a healthy offspring [8, 9]. It is most likely that completely immotile spermatozoa are often caused by genetic and pathological factors. However, it is not clear whether completely immotile spermatozoa can survive the freezing process. As previously reported, it is usual to use motile sperm for freezing in most IVF centers of worldwide. Hence sperm motility rates are most commonly used to evaluate the freezing effectiveness [24, 25]. In the present study, none of the spermatozoa were motile before freezing, laser assessment of viability was performed to detect whether these spermatozoa samples were suitable for cryopreservation, and the survival rates were also assessed by laser analysis after thawing.

Our study showed that the viable sperm in completely immotile sperm samples, including both those from ejaculates and of testicular origin, were able to survive the cryodamage during the freezing process. This resulted in a survival index of 0.80 and 0.70, which was similar to that obtained for ejaculated motile sperm [26, 27]. These live, viable but immotile spermatozoa after cryopreservation were selected for ICSI by laser analysis in order to confirm whether they still possessed the ability to fertilize normally. The results show 80% and 75% fertilization rate which was similar to that obtained from fresh spermatozoa [28, 29].

The key to explore the value of cryopreservation of complete immotile spermatozoa is how to identify and select viable spermatozoa before and after cryopreservation for ICSI. The latest manual of the World Health Organization, published in 2010, for examination and processing of human semen recommends the eosin test, which may be used in combination with negrosin and the HOS test for the diagnostic evaluation of viability [20]. The eosin test is the standard technique for determining sperm viability and is based upon the ability of viable spermatozoa to exclude eosin entering cells due to the integrity of their membrane structures, whereas dead spermatozoa will take up the dye and hence be stained. However, once exposed to such toxic dyes, spermatozoa are no longer available for ICSI [30], which limits the application of eosin in ART.

The HOS test has been reported to be successful in fertility treatment of patients with fresh completely immotile spermatozoa, and reasonable fertilization rates and clinical outcomes were achieved after ICSI [31]. But it must be noted that spontaneously developed tail swellings may influence the accuracy of the HOS-test in determining membrane integrity and viability of human spermatozoa [32]. Therefore, this method is not suitable for spermatozoa that have been processed, specifically for frozen-thawed spermatozoa. For the past few years, laser analysis has been widely used in the field of assisted reproduction, including assisted hatching, embryo biopsy and spermatozoa immobilization and permeabilization [19, 33, 34]. A healthy baby was delivered after laser assisted immobilization of spermatozoa before ICSI [35]. Furthermore, studies confirmed that the use of lasers for the treatment of spermatozoa is regarded as safe [36]. A viable spermatozoon is considered to be one with membrane integrity and capable of protein activation and a single laser shot applied to the far end of the flagellum of a viable spermatozoon caused a curling as was observed in positive HOS reactions. This characteristic provided a new criterion for embryologists to select viable but immotile spermatozoa. Successful pregnancies had previously been achieved by using laser assisted selection of viable but immotile sperm before ICSI [18, 37]. However, whether this method is suitable for identification and selection of completely immotile sperm after thawing was unclear. In the current study, we found that use of a laser was also an effective method for selection of viable frozen-thawed sperm for subsequent ICSI. Moreover, laser treatment can be easily carried out within normal culture medium and any selected spermatozoon can be used directly for ICSI, and this negates the use of any chemical reagent.

Nevertheless, this study has some limitations. First, because limitation of donated mature oocytes, only a portion of frozen-thawed sperm samples can be used to fertilization and embryo development verification. This may lead to a challenge to statistical analysis. Second, the research of male fertility preservation is mostly based on survival rate, fertilization rate and early embryo development competence, so large-scale clinical application are required to investigate the clinical outcome and health of offspring.

## Conclusions

Our study confirms that completely immotile spermatozoa including ejaculated and testicular derived sperm, combining with laser assisted selection of viable sperm, were able to be cryopreserved for further ART treatment as long as only viable but immotile sperm were used. This procedure can provide a new way of fertility preservation for patients with completely immotile spermatozoa.

## Abbreviations

ART: Assisted reproductive technology
HOS: Hypo-osmotic swelling
ICSI: Intracytoplasmic sperm injection
IVF: In vitro fertilization
PESA: percutaneous epididymal sperm aspriration
SPSS: Statistical Package for Social Sciences
TESA: Testicular aspiration
WHO: World Health Organization

## References

[1] Ron-El R, Raziel A, Strassburger D, Schachter M, Bern O, Friedler S (2000). Birth of healthy male twins after intracytoplasmic sperm injection of frozen-thawed testicular spermatozoa from a patient with nonmosaic Klinefelter syndrome. Fertil Steril.

[2] Matsumoto Y, Goto S, Hashimoto H, Kokeguchi S, Shiotani M, Okada H (2010). A healthy birth after intracytoplasmic sperm injection using ejaculated spermatozoa from a patient with Kartagener's syndrome. Fertil Steril.

[3] Geber S, Lemgruber M, Taitson PF, Valle M, Sampaio M (2012). Birth of healthy twins after intracytoplasmic sperm injection using ejaculated immotile spermatozoa from a patient with Kartagener's syndrome. Andrologia.

[4] Montjean D, Courageot J, Altie A, Amar-Hoffet A, Rossin B, Geoffroy-Siraudin C, Tourame P, Boyer P (2015). Normal live birth after vitrified/warmed oocytes intracytoplasmic sperm injection with immotile spermatozoa in a patient with Kartagener's syndrome. Andrologia.

[5] Stalf T, Mehnert C, Hajimohammad A, Manolopoulos K, Shen Y, Schuppe HC, Diemer T, Schill WB, Weidner W, Tinneberg HR (2005). Influence of motility and vitality in intracytoplasmic sperm injection with ejaculated and testicular sperm. Andrologia.

[6] Esfandiari N, Javed MH, Gotlieb L, Casper RF (2005). Complete failed fertilization after intracytoplasmic sperm injection--analysis of 10 years’ data. Int J Fertil Womens Med.

[7] Javed M, Esfandiari N, Casper RF (2010). Failed fertilization after clinical intracytoplasmic sperm injection. Reprod BioMed Online.

[8] McLachlan RI, Ishikawa T, Osianlis T, Robinson P, Merriner DJ, Healy D, de Kretser D, O'Bryan MK (2012). Normal live birth after testicular sperm extraction and intracytoplasmic sperm injection in variant primary ciliary dyskinesia with completely immotile sperm and structurally abnormal sperm tails. Fertil Steril.

[9] Nordhoff V, Schuring AN, Krallmann C, Zitzmann M, Schlatt S, Kiesel L, Kliesch S (2013). Optimizing TESE-ICSI by laser-assisted selection of immotile spermatozoa and polarization microscopy for selection of oocytes. Andrology.

[10] Philippon M, Karsenty G, Bernuz B, Courbiere B, Brue T, Saias-Magnan J, Perrin J (2015). Successful pregnancies and healthy live births using frozen-thawed sperm retrieved by a new modified Hotchkiss procedure in males with retrograde ejaculation: first case series. Basic Clin Androl.

[11] Sokmensuer LK, Bozdag G, Esinler I, Sever A, Gunalp S (2015). Live birth after transfer of vitrified-warmed blastocyst derived from ICSI with frozen-thawed sperm: case report. Clin Exp Obstet Gynecol.

[12] Cayan S, Lee D, Conaghan J, Givens CA, Ryan IP, Schriock ED, Turek PJ (2001). A comparison of ICSI outcomes with fresh and cryopreserved epididymal spermatozoa from the same couples. Hum Reprod.

[13] Sukcharoen N, Sithipravej T, Promviengchai S, Chinpilas V, Boonkasemsanti W (2001). Comparison of the outcome of intracytoplasmic sperm injection using fresh and frozen-thawed epididymal spermatozoa obtained by percutaneous epididymal sperm aspiration. J Med Assoc Thail.

[14] Wald M, Ross LS, Prins GS, Cieslak-Janzen J, Wolf G, Niederberger CS (2006). Analysis of outcomes of cryopreserved surgically retrieved sperm for IVF/ICSI. J Androl.

[15] Karacan M, Alwaeely F, Erkan S, Cebi Z, Berberoglugil M, Batukan M, Ulug M, Arvas A, Camlibel T (2013). Outcome of intracytoplasmic sperm injection cycles with fresh testicular spermatozoa obtained on the day of or the day before oocyte collection and with cryopreserved testicular sperm in patients with azoospermia. Fertil Steril.

[16] Hessel M, Robben JC, D'Hauwers KW, Braat DD, Ramos L (2015). The influence of sperm motility and cryopreservation on the treatment outcome after intracytoplasmic sperm injection following testicular sperm extraction. Acta Obstet Gynecol Scand.

[17] Tongdee P, Sukprasert M, Satirapod C, Wongkularb A, Choktanasiri W (2015). Comparison of cryopreserved human sperm between ultra rapid freezing and slow programmable freezing: effect on motility, morphology and DNA integrity. J Med Assoc Thail.

[18] Gerber P, Kruse R, Hirchenhain J, Krussel JS, Neumann NJ (1826). Pregnancy after laser-assisted selection of viable spermatozoa before intracytoplasmatic sperm injection in a couple with male primary cilia dyskinesia. Fertil Steril.

[19] Montag M, Rink K, Delacretaz G, van der Ven H (2000). Laser-induced immobilization and plasma membrane permeabilization in human spermatozoa. Hum Reprod.

[20] WHO: World Health Organization (WHO) (2010). Laboratory manual for the examination and processing of human semen.

[21] Aktan TM, Montag M Fau - Duman S, Duman S Fau - Gorkemli H, Gorkemli H Fau - rink K, rink K Fau - Yurdakul T, Yurdakul T. Use of a laser to detect viable but immotile spermatozoa. Andrologia 2004;36**:**366–369. 10.1111/j.1439-0272.2004.00636.x 15541052

[22] Desch L, Bruno C, Herbemont C, Michel F, Bechoua S, Girod S, Sagot P, Fauque P (2015). Impact on ICSI outcomes of adding 24 h of in vitro culture before testicular sperm freezing: a retrospective study. Basic Clin Androl.

[23] Alpha Scientists in Reproductive M, Embryology ESIGo. The Istanbul consensus workshop on embryo assessment: proceedings of an expert meeting. Hum Reprod. 2011;26**:**1270–1283. 10.1093/humrep/der037 21502182

[24] Liu F, Zou SS, Zhu Y, Sun C, Liu YF, Wang SS, Shi WB, Zhu JJ, Huang YH, Li Z. A novel micro-straw for cryopreservation of small number of human spermatozoon. Asian J Androl. 2017;19(3):326-29. 10.4103/1008-682X.173452 PMC5427789 26841935

[25] Yokonishi T, Ogawa T (2016). Cryopreservation of testis tissues and in vitro spermatogenesis. Reprod Med Biol.

[26] Stein A, Shufaro Y, Hadar S, Fisch B, Pinkas H (2015). Successful use of the Cryolock device for cryopreservation of scarce human ejaculate and testicular spermatozoa. Andrology.

[27] Ishikawa T, Shiotani M, Izumi Y, Hashimoto H, Kokeguchi S, Goto S, Fujisawa M (2009). Fertilization and pregnancy using cryopreserved testicular sperm for intracytoplasmic sperm injection with azoospermia. Fertil Steril.

[28] Park YS, Kim MK, Lim CK, Lee SH, Park DW, Seo JT, Yang KM (2014). Efficacy of cryopreservation of embryos generated by intracytoplasmic sperm injection with spermatozoa from frozen testicular tissue. J Assist Reprod Genet.

[29] Wu B, Wong D, Lu S, Dickstein S, Silva M, Gelety TJ (2005). Optimal use of fresh and frozen-thawed testicular sperm for intracytoplasmic sperm injection in azoospermic patients. J Assist Reprod Genet.

[30] Buckett WM (2003). Predictive value of hypo-osmotic swelling test to identify viable non-motile sperm. Asian J Androl.

[31] Tubman A, Check JH, Bollendorf A, Wilson C (2013). Effect of poor motility on pregnancy outcome following intracytoplasmic sperm injection in couples whose male partners have subnormal hypo-osmotic swelling test scores. Clin Exp Obstet Gynecol.

[32] Hossain A, Osuamkpe C, Hossain S, Phelps JY (2010). Spontaneously developed tail swellings (SDTS) influence the accuracy of the hypo-osmotic swelling test (HOS-test) in determining membrane integrity and viability of human spermatozoa. J Assist Reprod Genet.

[33] Blessmann-Roset J, Rives N, Clavier B, Milazzo JP, Mazurier S, Mousset-Simeon N, Mace B (2009). Laser assisted hatching: Rouen University Hospital outcomes. Gynecol Obstet Fertil.

[34] Rubino P, Cotarelo RP, Alteri A, Rega E, Vigano P, Giannini P (2014). Trophectoderm biopsy performed by a double laser zona drilling: first safety evidence. Eur J Obstet Gynecol Reprod Biol.

[35] Ebner T, Moser M, Yaman C, Sommergruber M, Tews G (2002). Successful birth after laser assisted immobilization of spermatozoa before intracytoplasmic injection. Fertil Steril.

[36] Germond M, Nocera D, Senn A, Rink K, Delacretaz G, Fakan S (1995). Microdissection of mouse and human zona pellucida using a 1.48-microns diode laser beam: efficacy and safety of the procedure. Fertil Steril.

[37] Chen HH, Feng GX, Zhang B, Shu JH, Gan XY, Zhou H, Lin RY (2015). Successful pregnancy following laser-assisted selection of viable but immotile spermatozoa for intracytoplasmic sperm injection: A report of 2 cases. Zhonghua Nan Ke Xue.

